# Sodium halide solid state electrolyte of Na_3_YBr_6_ with low activation energy

**DOI:** 10.1039/d4ra02663b

**Published:** 2024-05-07

**Authors:** Xiang-Yu Niu, Xin-Yi Dou, Cheng-Yu Fu, Yi-Chun Xu, Xu-Yong Feng

**Affiliations:** a School of Materials Science and Engineering, Anhui Provincial Key Laboratory of Advanced Functional Materials and Devices, Hefei University of Technology Hefei 230009 Anhui P. R. China 2021800026@hfut.edu.cn; b Key Laboratory of Materials Physics, Institute of Solid-State Physics, Chinese Academy of Sciences P. O. Box 1129 Hefei 230031 P. R. China xuyichun@issp.ac.cn

## Abstract

Halide solid-state electrolytes (SSEs) are considered promising candidates for practical applications in all-solid-state batteries (ASSBs), due to their outstanding high voltage stability and compatibility with electrode materials. However, Na^+^ halide SSEs suffer from low ionic conductivity and high activation energy, which limit their applications in sodium all-solid-state batteries. Here, sodium yttrium bromide solid-state electrolytes (Na_3_YBr_6_) with a low activation energy of 0.15 eV is prepared *via* solid state reaction. Structure characterization using X-ray diffraction reveals a monoclinic structure (*P*2_1_/*c*) of Na_3_YBr_6_. First principle calculations reveal that the low migration activation energy comes from the larger size and vibration of Br^−^ anions, both of which expand the Na^+^ ion migration channel and reduce its activation energy. The electrochemical window of Na_3_YBr_6_ is determined to be 1.43 to 3.35 V *vs.* Na/Na^+^, which is slightly narrower than chlorides. This work indicates bromides are a good catholyte candidate for sodium all solid-state batteries, due to their low ion migration activation energy and relatively high oxidation stability.

## Abstract 2

Vibration of Br^−^ during the Na^+^ ion migration can significantly reduce ion migration activation energy. Therefore, compared to chlorides, bromide should be able to achieve lower migration activation energy and higher Na^+^ ion conduction.

## Graphical abstract

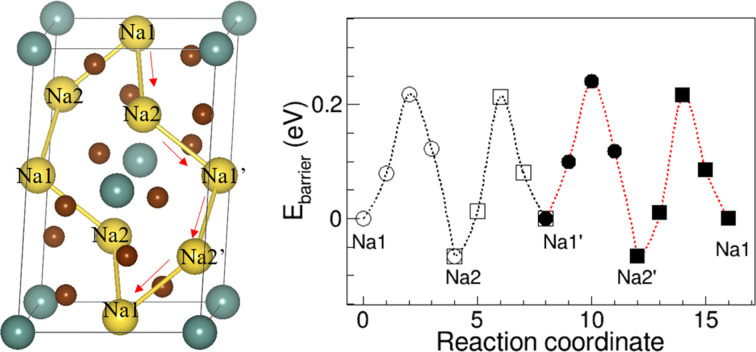

## 1. Introduction

All solid-state batteries (ASSBs) have attracted much attention in the past decades, due to their high safety and high energy density.^1–6^ The main difference between traditional batteries and ASSBs is the use of solid-state electrolytes (SSEs) to replace organic liquid electrolytes, which brings higher safety and better stability. However, SSEs also have new technique and economic issues, which hinder the commercialization of ASSBs. For example, the contact between SSEs and solid electrodes is poor, leading to high interface resistance. Furthermore, this contact would be even worse with volume change of electrodes during cycling, making ASSBs decay fast.^2,7–9^ Also, the Li^+^ ion concentration is always ten times higher than in liquid electrolytes, which increases the cost of ASSBs obviously.

Sodium has a similar ionic radius and potential, but is more abundant than lithium. The replacement of Li with Na can significantly reduce the cost while the electrochemical performance can be maintained to a large extent.^10–14^ However, similar technical issues still exist in Na based ASSBs. Since these issues are caused by the use of SSEs, developing high-performance SSEs is expected to solve these issues. In the past decades, several types Na ion SSEs have been developed and applied in Na ASSBs, such as oxides (NASICON, *etc.*),^15–19^ sulfides (Na_3_PS_4_, *etc.*),^20–23^ halides (Na_2_ZrCl_6_),^13,24,25^ borides^26–30^ and polymers.^31^ However, these electrolytes all have certain issues. For example, polymers are soft and easy to process, posing good compatibility to electrode materials and low cost, but the ionic conductivity is poor at room temperature.^31^ Sulfides have high ionic conductivity up to 10^−2^ S cm^−1^, which is comparable or even higher than liquid electrolytes, but the chemical and electrochemical stability is poor, leading to poor cycle performance when combining with high voltage cathodes.^14^ Oxides and halides are both stable against high voltage cathodes, but the rigid feature of oxides makes the poor contact to electrodes,^32^ while the ionic conductivity of halides is poor.^33,34^ The most reported sodium halide SSEs are chlorides, such as Na_3_YCl_6_,^35^ Na_2_ZrCl_6_,^13,25^ NaAlCl_4_ (ref. 33) *etc.* In such materials, the diffusion bottleneck is too small for Na^+^ ion diffusion, leading to high activation energy and low ionic conductivity at room temperature. To broaden the diffusion bottleneck and enhance the ion conduction, anions with bigger size can be applied, such as Br^−^, I^−^*etc.* Considering the oxidation stability, Br^−^ would be a good option for Na halide SSEs. Several bromides have been predicted to have higher ionic conductivity, such as Na_3_YBr_6_, Na_3_MBr_6_*etc.*^35–37^ But little experimental results have proved this.

In this paper, Na_3_YBr_6_ with space group of *P*2_1_/*c* was synthesized *via* solid state reaction, which shows different ion conduction characteristics to previous simulation results. A much lower ionic conductivity (4.57 × 10^−8^ S cm^−1^) combined relatively low activation energy (0.15 eV) was achieved. This indicates the presence of fast ion migration paths in Na_3_YBr_6_, but the mobility of Na^+^ ions is poor. Theoretical simulations indicate this low migration activation energy is due to the vibration of Br^−^ ions during Na^+^ ion migration, which further broadens the size of diffusion bottleneck and enables a fast Na ion migration path. Further enhancement in ionic conductivity can be achieved by increasing the mobility of Na^+^ ions, such as local structure rearrangement. Cyclic voltammetry test proves a relatively high oxidation stability up to 3.3 V *vs.* Na/Na^+^ for Na_3_YBr_6_.

## 2. Experiments

The raw materials of NaBr, YBr_3_ was purchased from Aladdin and used without further purification. Stoichiometric NaBr and YBr_3_ were weighed and hand milled for around 30 min to obtain an even mixture. This mixture was further sintered in a sealed quartz tube at different temperatures for 10 h to obtain sintered Na_3_YBr_6_. Then the sample with highest ionic conductivity was further ball milled at 500 rpm for another 10 h to optimize the structure and ionic conductivity.

Na_2.9_PS_3.9_Cl_0.1_ was prepared according to our previous report.^23^ The raw materials of Na_2_S, P_2_S_5_ and NaCl purchased from SINOPHARM was mixed according to stoichiometric ratio and ball milled at 500 rpm for 10 h. Then the mixture was sintered at 420 °C for 10 h to obtain final products.

The structure and phase purity were characterized with powder X-ray diffraction (PXRD). The as-sintered powders were placed on a glass holder, flatted and covered by a thin Kapton film to ensure an inert environment. The PXRD patterns were acquired on a PANalytical X'Pert PRO powder X-ray diffractometer at 45 kV and 40 mA at room temperature by using a Cu Kα radiation (*λ* = 1.5406 Å) with a fixed scanning speed of 2° min^−1^ from 10° to 80°.

The ionic conductivities and activation energies of Na_3_YBr_6_ samples were measured with Ac impedance spectroscopy (EIS) at variable temperatures, in the frequency range from 1 MHz to 1 Hz and a potential perturbation of 50 mV (Biologic SP-200). The as-prepared Na_3_YBr_6_ samples were cold pressed under a pressure of 300 MPa into pellets with a diameter of 12 mm and thickness of around 1 mm. The pellets were sandwiched by two steel rods for EIS measurements. The electronic conductivity was also measured using the same cell, with applying a voltage of 0.5 V and collecting current till it is stable.

The electrochemical stability of Na_3_YBr_6_ was tested with linear scanning voltammetry. The Na_3_YBr_6_ sample was hand milled with 30 wt% Super P and used as the cathode, Na_2.9_PS_3.9_Cl_0.1_ (ref. 23 and 38) was used as solid-state electrolyte and Na was used as anode. These three layers were pressed under 300 MPa in a cylinder cell respectively, with a diameter of 12 mm. The mass of different layers was 10 mg (Na_3_YBr_6_), 100 mg (Na_2.9_PS_3.9_Cl_0.1_) and 200 mg (Na) respectively. The cells were charged to 5 V or discharged to 0 V *vs.* Na/Na^+^, with a scan rate of 1 mV s^−1^.

To get insights into the migration of ion migration in atomic level, density functional theory based first-principles calculations were performed by the Vienna *Ab initio* Simulation Package (VASP) code.^39^ The interaction between ion and electron is described by the projector augmented wave (PAW) method.^40^ The generalized gradient approximation (GGA) with Perdew–Burke–Ernzerhol (PBE) function^41^ was used for the exchange correlation energy. A plane wave energy cutoff of 520 eV with 8 × 6 × 4 *k*-point meshes are applied to optimized the structure of Na_3_YBr_6_ until the force convergence to less than 0.01 eV Å^−1^. Based on the optimized structure, the transition states and energy barriers of diffusion paths were calculated by the Climbing Image Nudged Elastic Band (CINEB) in the VTST package.^42,43^

## 3. Results and discussions

**Fig. 1:**
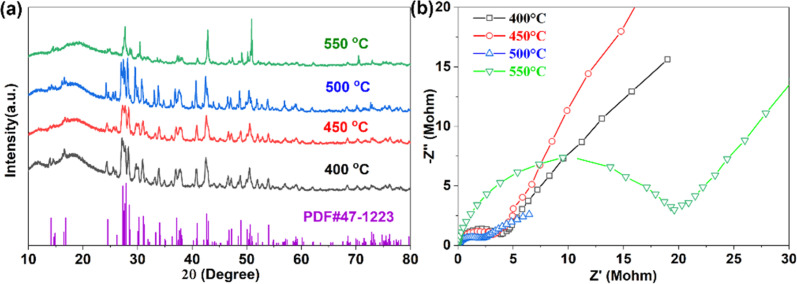
XRD pattern (a) and EIS (b) of Na_3_YBr_6_ sintered at different temperatures.

After high-temperature sintering, pure Na_3_YBr_6_ can be obtained, as shown in Fig. 1a. The XRD pattern for Na_3_YBr_6_ samples sintered between 400 °C and 500 °C are similar, all can be indexed to the pure Na_3_YBr_6_ with monoclinic structure (*P*2_1_/*c*). The lattice parameters are 7.17 Å (*a*), 7.67 Å (*b*), 12.84 Å (*c*) and 123.16° (*β*) (PDF# 47-1223). Comparing to Na_3_YCl_6_ (504.51 Å^3^, *Z* = 2),^44^ the unit cell volume of Na_3_YBr_6_ is bigger (591.22 Å^3^, *Z* = 2), indicating a larger size of Na^+^ ion migration path in Na_3_YBr_6_. When the temperature increases to 550 °C, the XRD changes significantly. For example, peaks around 27° emerge together and peak at 50° shifts and increases significantly, which indicates a structure change or decomposition of Na_3_YBr_6_.

The ionic conductivities of Na_3_YBr_6_ samples are calculated based on EIS (Fig. 1b), which shows only one clear semicircle. The grain boundary resistance maybe small and negligible. So we only consider the bulk resistance here. When temperature increases from 400 °C to 500 °C, the resistance of Na_3_YBr_6_ continuously decreases from 4 MΩ to about 2 MΩ, the corresponding ionic conductivity increases from 2.38 × 10^−8^ S cm^−1^ to 4.57 × 10^−8^ S cm^−1^. This indicates that Na_3_YBr_6_ powders are nonionic conductive, which is contradicting to the simulation results.^36^ As the temperature increases to 550 °C, the decomposition of Na_3_YBr_6_ (XRD pattern in Fig. 1a) leads to a significant decrease in its ionic conductivity to 5.10 × 10^−9^ S cm^−1^.

**Fig. 2:**
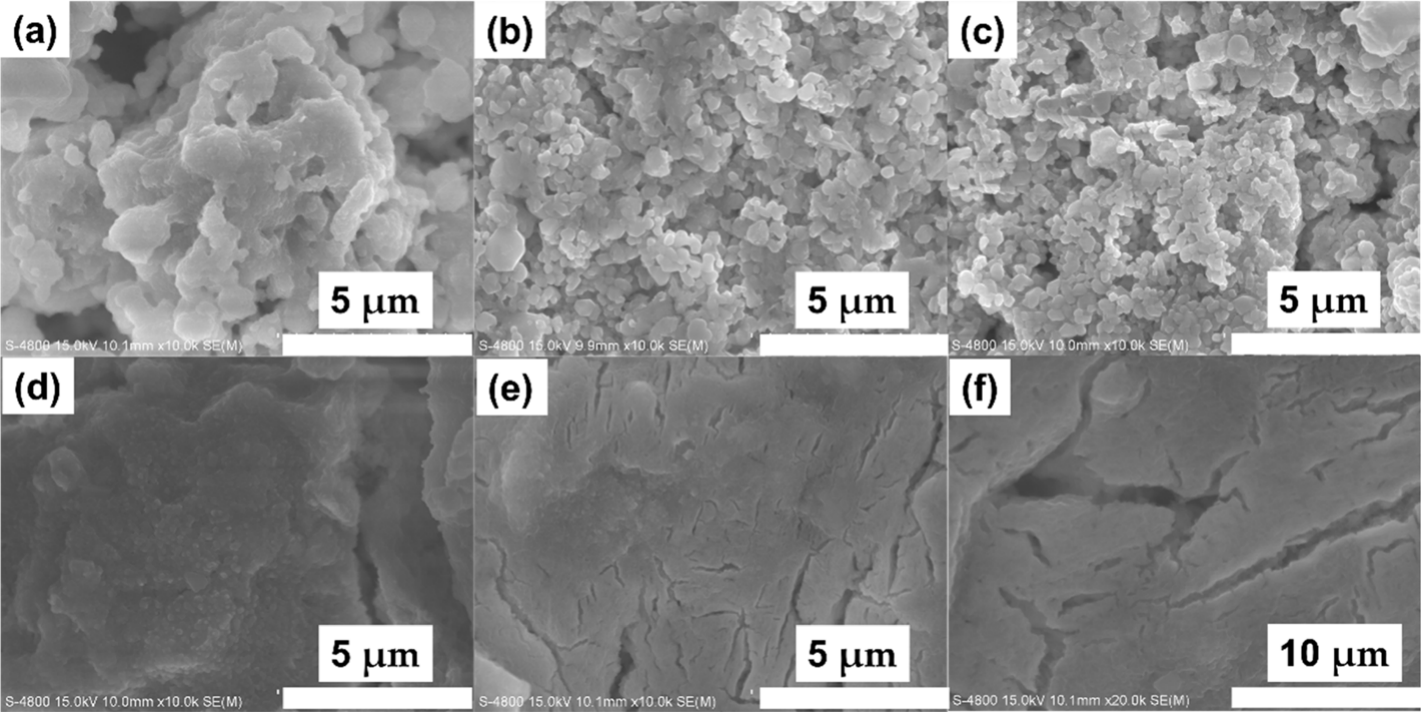
SEM images of Na_3_YBr_6_ sintered at 400 °C (a), 450 °C (b), 500 °C (c), 550 °C (d), and Na_3_YBr_6_ ball milled at 500 rpm after sintering at 500 °C (e and f).

SEM images of different samples are shown in Fig. 2. When sintered at 450 °C, the particles aggregate tightly. The irregular particle shape indicates the low crystallinity (Fig. 2a), which is consistent to the XRD pattern. When temperature increases, the particle size grows to hundreds of nanometers (Fig. 2b and c). When the temperature continues to rise to 550 °C, the decomposition of the product reduces the particle size, and the fast ion diffusion at high temperatures further aggregates the particles together (Fig. 2d).

**Fig. 3:**
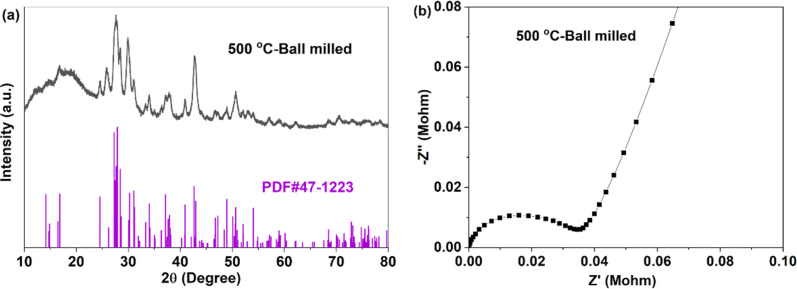
XRD pattern (a) and EIS (b) of Na_3_YBr_6_ sintered at 500 °C and ball milled at 500 rpm for 10 h.

For crystal materials with low ionic conductivity, local structure disordering after ball milling can significantly enhance the ionic conductivity, by changing the site energy and distribution of charge carriers at different sites.^5,45^ Here, the 500 °C sintered sample was further ball milled at 500 rpm for 10 h. After ball milling at 500 rpm for 10 h, the XRD pattern shows that the sample still maintains the Na_3_YBr_6_ structure, as shown in Fig. 3a. The difference is that the diffraction peaks become wider after ball milling, resulting in the adjacent peaks overlapping together. The broad peaks also indicate a decrease in crystal size and an increase in disordering in the Na_3_YBr_6_ sample. As shown in Fig. 2d and e, the particle size of Na_3_YBr_6_ significantly decreases after ball milling and these particles significantly agglomerate under pressure. Meanwhile, cracks between aggregates form, as shown in Fig. 2d and e. The tightly contact between small particles may help to reduce the grain boundary resistance and enhance the total ionic conductivity. Ionic conductivity can be significantly enhanced, from 4.57 × 10^−8^ S cm^−1^ to 2.84 × 10^−6^ S cm^−1^ (Fig. 3b), which is even higher than Na_3_YCl_6_ from literature.

**Fig. 4:**
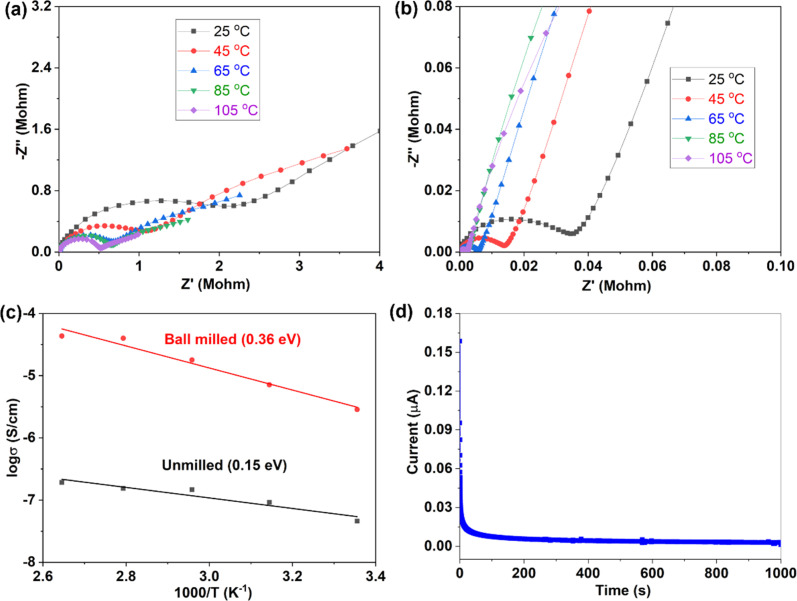
Variable temperature EIS of 500 °C samples before (a) and after (b) ball milling, (c) Arrhenius plots of 500 °C samples before and after ball milling, (d) DC polarization curve of 500 °C sample after ball milling.

Variable temperature EIS measurements were further conducted to obtain ion migration activation energy of different samples, as shown in Fig. 4. When temperature increases, the impedance decreases (Fig. 4a and b), indicating an increase of ionic conductivity. The relationship between ionic conductivity and temperature follows the Arrhenius formula 
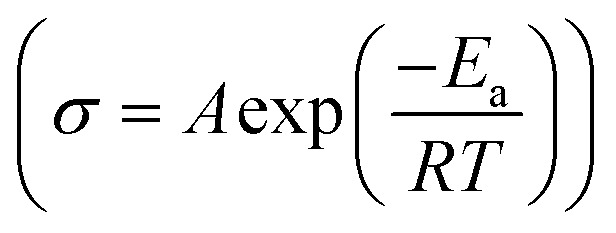
 within a certain temperature range. That is, the logarithm of ion conductivity is proportional to the reciprocal of temperature, and the slope is related to the migration activation energy. The activation energy can be calculated based on the Arrhenius plots (Fig. 4c), which is 0.15 eV for the 500 °C sample and 0.36 eV after ball milling. Although the activation energy of Na_3_YBr_6_ is low and comparable to other fast ion conductors. The pre-exponential factor A is much lower than other fast ion conductors, which is 3.7 × 10^−5^ for the 500 °C sample and 2.8 for the 500 °C sample after ball milling (Fig. 4c). This means Na^+^ ions in the Na_3_YBr_6_ sample are quite stable and have a low frequency to migrate to surrounding sites. As a result, the ionic conductivity of Na_3_YBr_6_ is still low (4.57 × 10^−8^ S cm^−1^) with such low activation energy (0.15 eV). According to the Arrhenius formula, the ionic conductivity is affected with two factors, pre-exponential factor A and activation energy *E*_a_. Here, the pre factor A is positively correlated with the concentration of charge carriers and the jumping frequency of charge carrier to the surrounding sites. The migration activation energy is related to the structure change along the migration path. The ball milling would change the local structure around Na^+^ ions, which reduces the stability of Na^+^ ions in Na_3_YBr_6_ and increase their jumping frequency to adjacent sites (A). Meanwhile, the distortion may increase the migration activation energy (*E*_a_). Here, the combined effect of both is an increase in ion conductivity, similar to the Ca^2+^ doped Na_3_PS_4_.^46^ Further enhancement in ionic conductivity can increase the site energy and thus migration frequency of Na^+^ ions through microstructure optimization, such as anion doping or lattice parameter adjustment. As solid-state electrolytes, low electronic conductivity is necessary. Here, the electronic conductivity was measured and the result is shown in Fig. 4d. It is calculated that the electronic conductivity of 500 °C sample after ball milling is about 4.2 × 10^−10^ S cm^−1^, which is four orders of magnitude lower than the ionic conductivity.

**Fig. 5:**
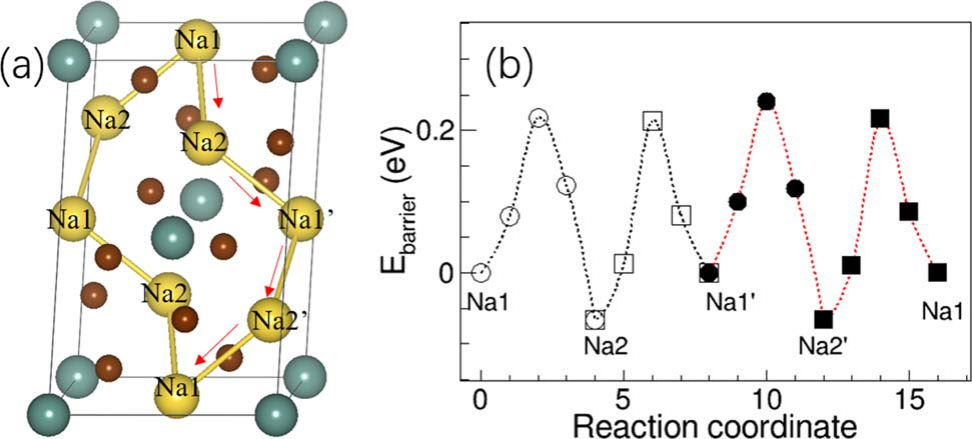
(a) The structure of Na_3_YBr_6_ and diffusion path of Na^+^, (b) the migration energy barrier of Na^+^ in the Na_3_YBr_6_.

Since the activation energy of Na_3_YBr_6_ measured here is much lower than the predicted value (∼0.43 eV) from BVSE method, which is much higher than the experimental results here (0.15 eV).^36^ Here, the relaxation of atoms next to Na^+^ during migration is considered, which should be closer to the actual migration process. The crystal structure of Na_3_YBr_6_ (monoclinic *P*21/*n*) was built according to the previous work^36^ and optimized to obtain the possible migration path way as shown in Fig. 5a. The energy barriers for Na^+^ migrating along the Na1–Na2–Na1′–Na2′–Na1 vary from 0.22 eV to 0.28 eV as shown in Fig. 5b, which is more consistent with the experimental results. Theoretical simulation results indicate that the relaxation of Br^−^ ions in bromide solid electrolytes plays an important role in reducing the activation energy of sodium ion migration and promoting the conduction of Na^+^ ions, which cannot be ignored in theoretical simulations.

**Fig. 6:**
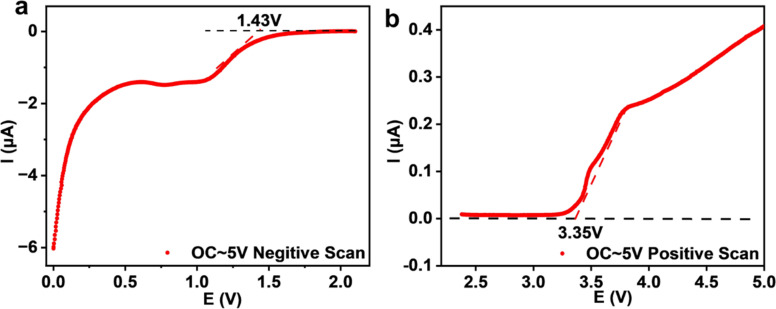
Linear scanning voltammetry of a Na|Na_2.9_PS_3.9_Cl_0.1_|Na_3_YBr_6_ + C cell.

The electrochemical stability of Na_3_YBr_6_ is also tested with linear scanning voltammetry and the result is shown in Fig. 6. To improve the activity of the Na_3_YBr_6_ sample and more accurately reflect its electrochemical stability, conductive carbon is added to Na_3_YBr_6_. When the anion changes from Cl^−^ to Br^−^ (Na_3_YCl_6_*vs.* Na_3_YBr_6_), the oxidation potential is supposed to be reduced. For Na_3_YBr_6_, it starts to oxidize at around 3.35 V *vs.* Na/Na^+^, which is comparable to theoretical results and lower than that of Na_3_YCl_6_.^35^ This is because in halides, oxidation reactions usually occur on anions, so Br^−^ with poor oxidation stability gives bromide (Na_3_YBr_6_) a lower oxidation potential to chlorides. The reduction potential of Na_3_YBr_6_ is simulated to be 0.57 V *vs.* Na/Na^+^.^35,36^ However, it is as high as 1.43 V *vs.* Na/Na^+^ according to our experimental results. This means the Na_3_YBr_6_ should be suitable for catholyte but not for anolyte.

## 4. Conclusions

In summary, Na_3_YBr_6_ with a low activation energy (0.15 eV) is prepared *via* solid state reaction. Structure characterizations reveal a monoclinic structure (*P*2_1_/*c*) of Na_3_YBr_6_, which has a bigger unit cell volume comparing to that of Na_3_YCl_6_. Experimental results show relatively low activation energy of 0.15 eV in sintered Na_3_YBr_6_ and 0.36 eV in ball milled Na_3_YBr_6_. Theoretical simulation results reveal that this low ion migration activation energy is due to the larger size of Br^−^ anion and the relaxation of Br^−^ anion during Na^+^ migration. The ionic conductivities of Na_3_YBr_6_ are measured to be 4.57 × 10^−8^ S cm^−1^ and 2.84 × 10^−6^ S cm^−1^ before and after ball milling, due to the low pre-exponential factor. Further enhancement in ionic conductivity can be achieved by local structure arrangement to increase the site energy and mobility of Na^+^ ions. The electronic conductivity of Na_3_YBr_6_ is measured to be 4.2 × 10^−10^ S cm^−1^, which is typically electronic insulator. Finally, the electrochemical stable window of Na_3_YBr_6_ is determined to be 1.43–3.35 V *vs.* Na/Na^+^, which should be stable against most cathode materials.

## Author contributions

X. F. and Y. X. conceived the research. X. N., X. D. and C. F. performed the materials synthesis, conductivity measurement, structural characterization and electrochemical tests. Y. X. conducted theoretical simulations. X. F. wrote the manuscript under the assistance of X. N., X. D., Y. X. and C. F.

## Conflicts of interest

There are no conflicts to declare.

## Supplementary Material
